# Mental health affects future employment as job loss affects mental health: findings from a longitudinal population study

**DOI:** 10.1186/1471-244X-13-144

**Published:** 2013-05-24

**Authors:** Sarah C Olesen, Peter Butterworth, Liana S Leach, Margaret Kelaher, Jane Pirkis

**Affiliations:** 1 Centre for Research on Ageing, Health & Wellbeing College of Medicine Biology & Environment, The Australian National University, Bldg 62A Eggleston Road, The Australian National University, Acton ACT 0200, Australia; 2 Melbourne School of Population and Global Health, Faculty of Medicine, Dentistry & Health Sciences, The University of Melbourne, Level 4 207 Bouverie Street, Carlton, Victoria 3010, Australia

**Keywords:** Mental health, Unemployment, Employment, Inclusion, Longitudinal, Social policy

## Abstract

**Background:**

Workforce participation is a key feature of public mental health and social inclusion policies across the globe, and often a therapeutic goal in treatment settings. Understanding the reciprocal relationship between participation and mental health has been limited by inadequate research methods. This is the first study to simultaneously examine and contrast the relative effects of unemployment on mental health and mental health on employment status in a single general population sample.

**Method:**

Data were from working-age respondents (20 to 55 years at baseline) who completed nine waves of the Household, Income and Labour Dynamics in Australia (HILDA) Survey (N=7176). Cross-lagged path analyses were used to test the lagged and concurrent associations between unemployment and mental health over time, adjusting for sociodemographic characteristics.

**Results:**

Mental health was shown to be both a consequence of and risk factor for unemployment. Thus, the poorer mental health observed amongst people who are not working is attributable to both the impact of unemployment and existing mental health problems. While the strength of these two effects was similar for women, the results for men suggested that the effect of unemployment on subsequent mental health was weaker than the effect of mental health on subsequent risk of unemployment.

**Conclusion:**

Disentangling the reciprocal links between mental health and workforce participation is central to the development and success of clinical goals and health and social policies that aim to promote either aspect. This study demonstrates that both effects are important and supports concurrent responses to prevent a cycle of disadvantage and entrenched social exclusion.

## Background

The benefits of workforce participation for mental health are established and routinely promoted in both clinical and health policy settings [1-3]. These benefits are believed to reflect a combination of material (e.g., income and the resulting access to resources) and psychological outcomes, such as social role and status, access to social networks and support, and a sense of purpose/achievement [4,5]. Conversely, mental *ill* health can be a barrier to gaining and maintaining employment [6-8]. These connections between mental health and economic participation are also at the forefront of public policy discussions beyond the health sector under the banner of social inclusion, a stated goal of many current governments [9-11]. ‘Inclusive’ societies and ‘included’ individuals are characterised by adequate social, educational, and economic participation. They demonstrate good health, wellbeing, and productivity [12,13]. In contrast, excluded individuals experience a set of multiple, and often entrenched, disadvantages including limited social support and networks, inadequate financial resources, and poor employment and health [14]. Policy attempts to bring about greater inclusiveness, harness the social and psychological benefits of workforce participation and improve employment opportunities amongst people who are unemployed or underemployed are still challenged by a lack of adequate epidemiological research into the reciprocal, causal links between participation and mental health [15].

The causal relationships between employment and mental health are yet to be disentangled in a single study. Cross-sectional research has established a contemporaneous link between unemployment and underemployment and poorer mental health [5] but cannot elucidate the temporal direction of this relationship [12]. Longitudinal studies have largely focused on the unidirectional effect of employment transitions on mental health [16-19], finding that job loss is related to a decline in men’s mental health [20]. Adverse mental health effects have also been shown in women who become unemployed or go on maternity leave [20]. Perhaps most relevant to the goals of clinicians and policy makers are the positive changes in mental health observed amongst people who return to work from unemployment [16,20].

The above studies provide a compelling argument that unemployment is a risk factor for psychological distress and potentially amenable to policy intervention. However they cannot exclude or qualify the role of a reciprocal relationship between employment and mental health; that is: how existing mental health problems impact upon job loss and gain [21,22]. A ‘health selection’ perspective on employment contends that physically and socially disabling aspects of illness can affect attendance and productivity at work [23-25]. This can in turn lead to employer- or employee-initiated job loss [26]. Consequently, people with mental health problems may also find themselves in a cycle of long-term unemployment; more likely to lose their job, and less able to seek future employment [27].

Fewer studies have examined the impact of mental health on employment using longitudinal data. Those that have tend to focus exclusively on people with a diagnosable mental disorder and find that this group is more likely to become unemployed compared to those without mental disorder [28,29] and that people with more severe symptoms are least likely to gain employment [30]. There is also some evidence for a link between continuous measures of psychological wellbeing and future employment. Mastekaasa [25] showed that high levels of distress are associated with greater risk of subsequent job loss relative to low distress. Kokko et al. [23] found that psychological distress during childhood is linked to longer-term unemployment in later life see also [31]. Together, these studies suggest that the impact of poor mental health on labour-force participation is not limited to severe mental illness.

The implications of mental ill-health as a consequence *versus* determinant of unemployment are quite different. For policy makers, the former typically invokes labour-market programs, participation requirements or financial incentives or disincentives. The latter requires efforts to identify and address barriers to employment and provide workplace accommodations for people with mental health problems. While there has been independent research into both causal pathways, no study has simultaneously assessed and compared these bi-directional effects in the same sample. This is a significant limitation in a research literature that cannot rule out cross-study inconsistencies due to sample and measurement differences. Further, unidirectional investigations cannot examine the relative importance of each causal pathway. That is, which pathway contributes most strongly to the observed association between poor mental health and unemployment, and thus, which should be the focus of clinical goals and policy efforts towards social inclusion.

The unique aim and contribution of this study is to simultaneously examine the two reciprocal associations between unemployment and poor mental health using a validated, continuous measure of psychological distress. We investigate and contrast: (i) the impact of unemployment on subsequent mental health and (ii) the effect of mental health on subsequent unemployment over nine waves of longitudinal data.

## Methods

### Study design and setting

Data were from nine waves of the Household, Income and Labour Dynamics in Australia (HILDA) Survey (release 9.0); a nationally representative household panel survey conducted annually from 2001. Participant consent was obtained and the HILDA survey was approved by the Human Research Ethics Committee at the University of Melbourne. The survey used a multi-stage sampling approach, sampling households within dwellings within a selection of administrative areas. At baseline, there were 7,682 responding households (response rate of 66%); including 13,969 household members aged 15 years and older (92% of the eligible population) who completed a personal interview. Ninety-four per cent of these respondents returned a self-completion questionnaire (SCQ) containing data on their mental health.

Given the focus on employment, analyses are restricted to working-age respondents (20 to 55 years at baseline) to minimise the influence of age-normative transitions into and out-of the workforce (e.g., retirement). This resulted in a sample of 8315 respondents. Over nine waves of data, the average wave-to-wave attrition in this subsample was 6.9%. The likelihood of attrition from the sample was not associated with baseline mental or physical health (10-point increase on Mental Health Inventory: OR = 0 .99, 0.96 - 1.01; 10-point increase on Physical Functioning scale: OR =0.98, 0.95 - 1.01), but was greater amongst those without a partner (OR = 1.06, 95% CI = 0.93 – 1.20), those who were unemployed (OR = 1.43, 1.15-1.78) and those otherwise not participating in the labour force (OR = 1.17, 1.03 – 1.32). As we were concerned with longitudinal analysis and cross-lagged predictors of mental health and unemployment, we further restricted the sample to those with three or more waves of data available for analysis. This resulted in a final sample size of 7176 cases (3371 men and 3805 women). Of these, 81.6% provided data at the final wave of data collection. Over the course of the study, 14.2% of these respondents reported at least one occasion of unemployment (n=1018, 13.6% of men and 14.7% of women). The majority of these respondents (69.4%) were identified as unemployed for only one wave of the survey.

The analytic strategic for this study uses all available data for the variables included in the model, with the weighted least squares estimator (similar to the maximum likelihood procedure) producing consistent and efficient estimators [32]. Only cases with missing data for baseline covariates were not able to be included in the models reported (that is, 60 men and 48 women). Thus, the sample sizes for the analyses reported in this paper range between 7068 and 7176.

### Measures

Mental health was assessed using the Mental Health Inventory (MHI-5), a subscale of the Short-Form Health Survey SF-[33,36] that was included in the SCQ at every wave of the HILDA Survey. The MHI-5 assesses symptoms of depression and anxiety (nervousness, depressed affect) and positive aspects of mental health (feeling calm, happy) during the past four weeks. Respondents indicate the frequency of these symptoms and responses are summed and transformed to a normalised scale from 0 to 100. Previous research has demonstrated that the MHI-5 is an effective screening tool for high-prevalence mental disorders (depression and anxiety disorders) in the general community [34,35]. Analysis of baseline HILDA Survey data showed adequate internal consistency (Cronbach’s alpha=0.82). The present analyses interpret the MHI-5 as a dimensional measure of these common mental health problems in light of the high comorbidity between symptoms of anxiety and depression [16] and evidence that internalising disorders reflect a higher-order factor [17]. Consistent with previous studies, we use the reversed scale where higher scores represent poorer mental health and greater distress.

Respondents were asked about their current labour-force status during each interview. This information was used to categorise respondents as working, unemployed but actively looking for work, or not participating in the work force (not in labour force; NILF) at each wave. Two dummy-coded variables were used to represent unemployment and NILF compared to employment. Periods of being NILF are not uncommon and differ to unemployment because individuals are not actively looking for work at these times, and are more likely to be *voluntarily* out of the labour force compared to periods of unemployment. As the focus of this study was to specifically investigate associations between mental health and unemployment (i.e., involuntary non-participation), NILF status was included a covariate in all analyses to control for these other periods of non-participation.

Time-invariant (baseline) covariates of age and sex were included in all models. Time-variant measures included NILF status (compared to unemployment), partner status (married/de facto [living with a partner] *versus* no partner) and physical functioning. The Physical Functioning subscale (PF-10) of the SF-36 assesses the degree of functional limitations caused by physical ill-health. In this analysis higher scores indicate poorer functioning (range 0–100).

### Analyses

**Figure 1:**
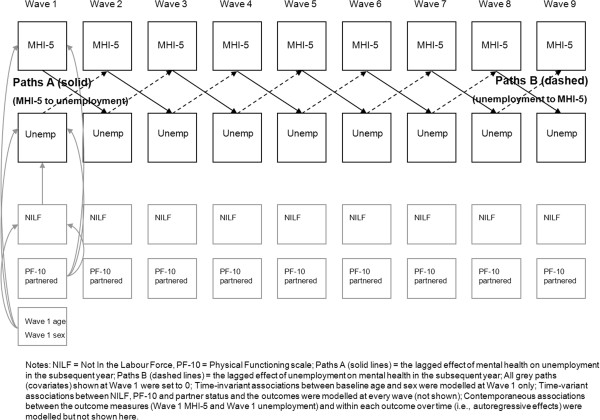
Simplified description of the 9-wave, cross-lagged model (not all paths shown).

Cross-lagged path analyses were conducted using Mplus (7.0) to simultaneously examine reciprocal, longitudinal relationships between unemployment (*versus* employment) and mental health while controlling for contemporaneous covariates. A simplified, conceptual version of the model used for our analyses is represented in Figure 1. As shown, the key associations between unemployment and mental health and vice versa were lagged in the model so that Paths A estimate the effect of mental health on subsequent unemployment (i.e., in the following interview) and Paths B estimate the effect of unemployment on subsequent mental health. The model assesses cross-lagged and autoregressive effects independent of contemporaneous associations between outcome measures at each wave, includes key time-varying and time-invariant covariates, and holds all paths consistent across waves (i.e., assumes the effects between mental health and unemployment are the same at each wave). The wave 1 variables age and sex are covariates. Preliminary analyses assessed the appropriateness of these assumptions through assessment of model fit and evaluated evidence of gender differences and the need for gender-specific models.

Models used the ‘weighted least-squares with mean and variance adjustment estimator’ due to the categorical nature of many of the variables [36]. Adequacy of model fit was assessed using: comparative fit index (CFI), Tucker-Lewis index (TLI) (>.95), and root mean square error of approximation (RMSEA; <0.07).

We assessed the relative impact of Paths A and B by removing each of these pathways. Difference tests were used to assess the significance of change in model fit upon these removals [37]. As such, Model 1 included all cross-lagged terms. Model 2 tested the contribution of Paths A by fixing this pathway to 0. Model 3 fixed the Paths B to 0. To evaluate the contribution of each pathway to overall model fit, Models 2 and 3 were each compared to Model 1. Model 4 fixed both Paths A and B to 0 and evaluated change from Models 2 and 3.

## Results

Baseline characteristics of the sample and correlations between the outcome measures across waves are presented in Tables 1 and 2 respectively. The results show a decline in the level of unemployment in the sample across waves, while mean MHI-5 scores remain stable over time. Table 2 shows that the correlations within measures decline with increasing distance between waves), that measures of unemployment and mental health are moderately correlated across waves, and that there is a consistent negative correlation between unemployment and mental health. The average correlation across all measures was *r*=−0.16.

**Table 1:** Characteristics of the HILDA sample used in the reported analyses

**Sociodemographics at Wave 1**		
Sex (male)		47.0%
Age (mean)		37.8 years
Partnered (married/de facto)		70.6%
Labour-force status	Employed	78.8% 4.2%
	Unemployed	47.0%
	Not in the labour force	17.0%
Outcome variables over time		
Wave	Mean MHI-5	% unemployed
1	73.4	4.2
**2**	73.7	3.6
**3**	74.0	2.7
**4**	73.9	2.4
**5**	73.9	2.4
**6**	74.3	2.5
**7**	74.2	2.1
**8**	74.4	2.0
**9**	74.8	2.4

**Table 2:** Correlations of outcome variables (MHI-5 and unemployment status) across 9 waves of HILDA data

**Unemployment polychoric correlations across waves**									
Unemployed Wave 1	1.00								
Unemployed Wave 2	0.66	1.00							
Unemployed Wave 3	0.44	0.53	1.00						
Unemployed Wave 4	0.37	0.37	0.57	1.00					
Unemployed Wave 5	0.42	0.34	0.49	0.55	1.00				
Unemployed Wave 6	0.35	0.40	0.50	0.48	0.60	1.00			
Unemployed Wave 7	0.32	0.34	0.48	0.40	0.45	0.58	1.00		
Unemployed Wave 8	0.29	0.39	0.46	0.45	0.52	0.60	0.65	1.00	
Unemployed Wave 9	0.37	0.33	0.40	0.39	0.40	0.52	0.19	0.66	1.00
**MHI-5 correlations across waves**									
MHI-5 Wave 1	1.00								
MHI-5 Wave 2	0.59	1.00							
MHI-5 Wave 3	0.54	0.62	1.00						
MHI-5 Wave 4	0.54	0.57	0.65	1.00					
MHI-5 Wave 5	0.51	0.53	0.59	0.64	1.00				
MHI-5 Wave 6	0.52	0.55	0.60	0.59	0.65	1.00			
MHI-5 Wave 7	0.49	0.55	0.59	0.58	0.62	0.67	1.00		
MHI-5 Wave 8	0.49	0.54	0.57	0.58	0.60	0.64	0.66	1.00	
MHI-5 Wave 9	0.50	0.53	0.54	0.55	0.56	0.60	0.61	0.66	1.00
	Unemployment								
	Wave 1	Wave 2	Wave 3	Wave 4	Wave 5	Wave 6	Wave 7	Wave 8	Wave 9
MHI-5 Wave 1	−0.17	−0.22	−0.15	−0.25	−0.11	−0.20	−0.07	−0.09	−0.14
MHI-5 Wave 2	−0.09	−0.23	−0.14	−0.23	−0.16	−0.20	−0.13	−0.20	−0.07
MHI-5 Wave 3	−0.10	−0.21	−0.16	−0.24	−0.11	−0.22	−0.15	−0.17	−0.11
MHI-5 Wave 4	−0.13	−0.17	−0.10	−0.22	−0.15	−0.21	−0.12	−0.17	−0.04
MHI-5 Wave 5	−0.15	−0.22	−0.15	−0.17	−0.15	−0.22	−0.11	−0.17	−0.14
MHI-5 Wave 6	−0.11	−0.21	−0.12	−0.17	−0.13	−0.24	−0.17	−0.17	−0.11
MHI-5 Wave 7	−0.11	−0.16	−0.12	−0.22	−0.13	−0.20	−0.17	−0.15	−0.07
MHI-5 Wave 8	−0.13	−0.19	−0.10	−0.19	−0.12	−0.20	−0.13	−0.21	−0.06
MHI-5 Wave 9	−0.16	−0.20	−0.12	−0.17	−0.13	−0.17	−0.18	−0.16	−0.15

### Preliminary models

An initial set of models evaluated the consistency of results between men and women, contrasting the models (the simple model including only mental health and labour-force variables, and the full model with covariates) where paths were held the same for men and women with models where these paths were able to differ by gender. The results for both the simple (chi square = 272.90, df = 11, p < .001) and multivariable models (chi square = 1223, df = 19, p < .001) suggested that the association between employment status and mental health differed for men and women. Therefore, gender specific models are reported in the remainder of this analysis.

### Final models

Model fit and model comparison statistics are presented in Table 3 for men and women separately.

**Table 3:** Model fit statistics for cross-lagged path models of unemployment and mental health

**Men**	**CFI**	**TLI**	**RMSEA**	**Change χ**^**2**^	**df**	**p**
Model 1 both lagged effects	0.93	0.93	0.046			
Model 2 no lagged effect of MHI-5 (no Path A)	0.93	0.93	0.046	16.57ª	1	<.0001
Model 3 no lagged effect of unemployment (no Path B)	0.93	0.93	0.0436	4.15ª	1	=.042
Model 4 neither lagged effect	0.93	0.93	0.043			
no lagged effect of unemployment: compared to Model 2				9.03	1	=.0027
no lagged effect of MHI-5: compared to Model 3				16.81	1	<.0001
**Women**						
Model 1 both lagged effects	0.95	0.95	0.043			
Model 2 no lagged effect of MHI-5 (no Path A)	0.95	0.95	0.044	38.51ª	1	< .0001
Model 3 no lagged effect of unemployment (no Path B)	0.95	0.95	0.043	19.05ª	1	< .0001
Model 4 neither lagged effect	0.95	0.95	0.044			
no lagged effect of unemployment: compared to Model 2				32.54	1	< .0001
no lagged effect of MHI-5 : compared to Model 3				50.63	1	< .0001

ª compared to Model 1.

For men (upper panel of Table 3), Model 1 represented an adequate fit to the data (though the CFI/TFI was slightly below 0.95). The cross-lagged coefficients provide evidence for the significance of Path A (the effect of mental health on unemployment) and Path B (the effect of unemployment on mental health). The coefficients representing Paths A (z=−3.76) and Paths B (z=−2.18) were both significant, indicating the presence of both effects, simultaneously, during the observation period. A significant contemporaneous association between unemployment and mental health was also evident (z=−2.87), indicating a relationship between these two factors within the same wave of data collection.

Removing the cross-lagged pathway leading from mental health to subsequent unemployment (Path A) significantly reduced the overall fit of the model (evident by the significant change is chi-square for Model 2). Similarly, removing the cross-lagged pathway leading from unemployment to subsequent mental health (Path B) significantly reduced overall model fit (Model 3), although this effect was less pronounced. Two further analyses examined the contribution of Paths A and B to these reduced models through comparison with a model in which pathway neither was included (see comparisons with Model 4 in Table 3). Again, the exclusion of each of the cross-lagged pathways further reduced overall model fit, with the deletion of Path A (from mental health to unemployment) again producing the greatest effect. Taken together, these results indicate that the poorer mental health observed amongst men who are unemployed (*versus* employed) is attributable to both the impact of unemployment on mental health and mental health on subsequent employment. However, the lagged effect of mental health on unemployment is somewhat stronger.

For women (lower panel, Table 3), the initial model fit was also adequate. Both the cross-lagged pathways from mental health to subsequent unemployment (z=−6.46) and from unemployment to subsequent mental health (z=−4.88) were significant and similar. The contemporaneous association between unemployment and mental health was also significant (z=−3.33). Further, the removal of each of these pathways at the second step of the modelling process significantly reduced overall model fit. While both pathways (from unemployment to subsequent mental health, and from mental health to subsequent unemployment) contributed significantly to the overall model fit, the results suggest that the pathway from mental health to subsequent unemployment was again somewhat stronger. This is consistent with the pattern of results evident for men.

## Discussion

Disentangling the relationship between mental health and unemployment provides an important evidence base to inform national health and social inclusion policies [12]. This is the first study to simultaneously examine and contrast the relative strengths of the bi-directional pathways between unemployment and mental health in a single population sample. Poor mental health was found to be both a consequence of and risk factor for unemployment in equal strengths, over and above the contemporaneous association observed between these two factors. The evidence for men in particular, suggested that mental health was a stronger predictor of subsequent unemployment than unemployment was a predictor of subsequent mental health.

Our finding that mental health is consistently associated with future job loss is consistent with previous research [8,22,38]. However, most studies that have linked mental illness to unemployment have focused on people with severe and low prevalent psychiatric disorders. This contrasts with our use of a continuous measure of mental health symptoms in a general community sample. Women typically report more depressive and anxiety symptoms than men [39], whilst men are known to demonstrate higher rates of low prevalence disorders [40]. Thus, the stronger associations evident for women in this sample may reflect the fact that men reported relatively better mental health (as defined by our measure) compared to women. The results of Prause and Dooley [16] and our previous research [41] offer some support for this explanation. Using a continuous measure of depression, Prause and Dooley found that men with low levels of depression were more likely to gain employment that their female counterparts but this advantage was less apparent amongst people with higher levels of depression. Using the first five waves of the HILDA Survey, we found that men’s mental health was associated with subsequent *duration* of unemployment but not increased *likelihood* of unemployment [41].

We also showed that unemployment was associated with poorer mental health in the future. However, this effect was somewhat weaker than the pathway in the reverse direction, particularly for men. We offer two possible explanations for this result. Firstly, we found that the experience of unemployment at baseline was associated with some increased likelihood of attrition from the survey. Explorative analysis showed that this effect was limited to men in the sample (men: OR = 1.52, 1.15 - 2.014; women: OR = 1.28, 0.90 - 1.81). Thus, we may have been less able to capture the lagged mental health consequences of unemployment due to greater sample attrition. Secondly, the effects of unemployment on mental health may be most evident more proximally to the actual event of unemployment than could be detected by the data collection method of the HILDA Survey. The waves of data for the HILDA Survey are collected, on average, 12 months apart. Thus, the time lag between waves may have somewhat masked the strength of this effect.

### Limitations and strengths

We acknowledge limitations to the generalisability of our findings. Due to the measures available in the HILDA Survey, we could not consider diagnosable mental illness. We instead focused on a continuous concept of mental health that is more sensitive to detecting change, including subclinical change [42]. Secondly, we restricted our analyses to respondents of prime working age (20 to 55 years at baseline) to avoid the influence of normative workforce transitions. Consequently, our results and recommendations should not be applied to younger and older cohorts without repeated studied in these age groups. The analytic technique used in this study is a significant strength that offers advantages over previous studies on this topic: chiefly and significantly, the ability to simultaneously test and compare the strength of bi-directional effects unemployment and mental health over time amongst the same individuals. Such evidence is essential to the formation of policies that deal with multi-faceted concepts (e.g., social inclusion) and aims (e.g., to improve both health and participation). The priority of this ‘in principle’ study was to clarify the bi-directional relationship between unemployment and mental health. Future research and additional modelling techniques will extend the current findings to elucidate the roles of additional socioeconomic and health factors that may contribute to this relationship.

### Implications for policy and practice

The findings of this study have clear implications for health and social inclusion policies in the general community and amongst people with existing mental health problems more specifically. Our findings provide strong support that, on average, the poorer mental health observed amongst people who are unemployed (*versus* employed) is attributable to both the impact of unemployment on (subsequent) mental health and mental health on (subsequent) unemployment. The occurrence of these two longitudinal effects alongside a strong contemporaneous association also supports the contention that poor mental health and non-participation have a cyclical relationship that may lead to entrenched disadvantage [12,14]. Accordingly, concurrent policies that aim to improve employment prospects for all people at risk of unemployment and, specifically, address the barriers to employment faced by people with existing mental health problems appear required. The latter aim is explicit in Australia’s current National Mental Health Plan [2], which is guiding the development of current and future policies in this sector. Our findings thus support for this priority and the continuation and expansion of interventions to assist people with mental health problems to remain in or re-enter the workforce [43] and programs to reduce mental health problems in the general community. However, for men, the pathway from unemployment to poor mental health was less consistent. Policies to promote and maintain workforce participation should be a focus of mental health prevention programs, particularly for men.

## Conclusions

This study demonstrates that poor mental health is both a consequence and determinant of unemployment, that the strength of each relationship is broadly consistent, but that gender differences are present. These findings highlight the cyclical and potentially entrenched nature of poor mental health and participation and the need for a similarly reciprocal design in employment, welfare, and mental health policies.

## Competing interests

The authors declare that they have no competing interests.

## Authors’ contributions

SCO drafted the manuscript, tables and figure. PB performed statistical analyses. All authors contributed to the conceptual design of the study, interpretation of the data, and revisions to the manuscript. All authors read and approved the final manuscript.

## Pre-publication history

The pre-publication history for this paper can be accessed here:

http://www.biomedcentral.com/1471-244X/13/144/prepub
